# Model-Agnostic Structural Transfer Learning for Cross-Domain Autonomous Activity Recognition

**DOI:** 10.3390/s23146337

**Published:** 2023-07-12

**Authors:** Parastoo Alinia, Asiful Arefeen, Zhila Esna Ashari, Seyed Iman Mirzadeh, Hassan Ghasemzadeh

**Affiliations:** 1 School of Electrical Engineering and Computer Science, Washington State University, Pullman, WA 99164, USA; parastoo.alinia@wsu.edu (P.A.); z.esnaashariesfahan@wsu.edu (Z.E.A.); seyediman.mirzadeh@wsu.edu (S.I.M.); 2 College of Health Solutions, Arizona State University, Phoenix, AZ 85004, USA; hassan.ghasemzadeh@asu.edu

**Keywords:** activity recognition, wearables, mobile health, machine learning, transfer learning, model-independent, structural similarity

## Abstract

Activity recognition using data collected with smart devices such as mobile and wearable sensors has become a critical component of many emerging applications ranging from behavioral medicine to gaming. However, an unprecedented increase in the diversity of smart devices in the internet-of-things era has limited the adoption of activity recognition models for use across different devices. This lack of cross-domain adaptation is particularly notable across sensors of different modalities where the mapping of the sensor data in the traditional feature level is highly challenging. To address this challenge, we propose *ActiLabel*, a combinatorial framework that learns structural similarities among the events that occur in a target domain and those of a source domain and identifies an optimal mapping between the two domains at their structural level. The structural similarities are captured through a graph model, referred to as the *dependency graph*, which abstracts details of activity patterns in low-level signal and feature space. The activity labels are then autonomously learned in the target domain by finding an optimal tiered mapping between the dependency graphs. We carry out an extensive set of experiments on three large datasets collected with wearable sensors involving human subjects. The results demonstrate the superiority of ActiLabel over state-of-the-art transfer learning and deep learning methods. In particular, ActiLabel outperforms such algorithms by average F1-scores of 36.3%, 32.7%, and 9.1% for cross-modality, cross-location, and cross-subject activity recognition, respectively.

## 1. Introduction

Smart devices such as wearable and mobile sensors are increasingly utilized for health monitoring and personalized behavioral medicine. These technologies use machine-learning/deep-learning algorithms to detect lifestyle and physiological biomarkers and to provide real-time clinical interventions [1,2,3,4,5,6,7]. However, the machine learning models are designed based on labeled training data collected in a particular domain, such as with a specific sensor modality, wearing site, or user. A significant challenge with this approach is that a machine learning model trained with a specific setting performs extremely poorly in new settings such as when the model is used with a sensor of a different modality, when the on-body location of the sensor changes, or when a new subject adopts the system [8,9]. This generalizability challenge has limited scalability of sensor-based health monitoring because collecting a sufficiently large number of labeled sensor data for every possible domain is a time-consuming, labor-intensive, expensive, and often infeasible process.

To address the aforementioned challenges, we introduce *ActiLabel*, a combinatorial framework that learns machine learning models in a new domain (i.e., target) without the need to manually collect any labels. Our pilot application in this paper is activity recognition, where ActiLabel is designed to detect human activities from wearable sensor data. A unique attribute of ActiLabel is that it examines structural relationships between activity events (i.e., classes/clusters) in the two domains and uses this information for target-to-source mapping. Such structural relationships allow us to compare the two domains at a much higher level of abstraction than the common feature space and therefore enable knowledge transfer across radically diverse domains. We hypothesize that even under sever cross-domain spatial and temporal uncertainties (i.e., significant distribution shift due to sensor modality change), physical activities exhibit similar structural dependencies across the two domains. We aim to uncover such structural dependencies from the sensor data gathered in the two domains and use this knowledge for mapping sensor data from the target domain to the data in the source domain.

To the best of our knowledge, our work is the first study that develops a combinatorial approach for structural transfer learning. Our notable contributions can be summarized as follows: (i) we introduce a model-agnostic combinatorial optimization formulation for transfer learning where no labeled data are available in the target domain, and we show that this problem is non-deterministic polynomial-time hardness (NP-hard); (ii) we devise methodologies for constructing a network representation of wearable sensor readings, referred to as *network graph*, as integral components of our framework for understanding structural dependencies among activity classes; (iii) we design algorithms that perform community detection on the network graph to identify core activity clusters; (iv) we introduce an approach to construct a dependency graph based on the core activity clusters identified on the network graph; (v) we show that combinatorial transfer learning can be transformed into a tractable assignment problem in the new knowledge transfer space given by the dependency graphs; (vi) we propose a novel multi-layer matching algorithm for mapping target-to-source dependency graphs; and (vii) we conduct an extensive assessment of the performance of ActiLabel for cross-modality, cross-subject, and cross-location activity learning using real sensor data collected with human subjects.

### 1.1. Transfer Learning

Transfer learning is the ability to extend what has been learned in one setting (i.e., source) to another, nonidentical but related, setting (i.e., target). Based on the common analogy in machine learning, we refer to the previous setting as the *source domain*. The sensor data captured in this domain is referred to as the source dataset, which is fully labeled in our case. The new state of the system, which may exhibit radical changes from the source domain, is referred to as the *target domain* where we intend to label the sensor data autonomously [10].

**Definition** **1.** *(Transfer Learning). Given a source domain $D_{s}$ and learning task $T_{s}$, a target domain $D_{t}$ and learning task $T_{t}$, transfer learning aims to help improve the learning of the target predictive function $F_{t}\left(.\right)$ in $D_{t}$ using the knowledge in $D_{s}$ and $T_{s}$, where $D_{s}=D_{t}$ or $T_{s}=T_{t}$.*

Depending on how the source and target tasks and domains are defined, one can categorize transfer learning techniques into inductive transfer learning, transductive transfer learning, and unsupervised transfer learning. Inductive transfer learning refers to the case where the tasks in the target and source are different. Therefore, we need some labeled data to induce a prediction model in the target domain. In transductive transfer learning, the source and target obtain the same tasks but different domains. In this setting, there is no label in the target but a relatively large amount of labeled data is available in the source domain. Finally, in the unsupervised transfer learning, the target task is different from but related to the source, and no label is available in the target domain. Unsupervised transfer learning aims to solve unsupervised learning problems such as clustering and dimensionality reduction [11,12]. The transductive transfer learning, which is the focus of this paper, can be defined as follows.

**Definition** **2.** *(Transductive Transfer Learning) Given a source domain $D_{s}$ and a corresponding learning task $T_{s}$, a target domain $D_{t}$ and a corresponding learning task $T_{t}$, transductive transfer learning aims to improve the learning of the target predictive function $F_{t}\left(.\right)$ in $D_{t}$ using the knowledge in $D_{s}$ and $T_{s}$, where $D_{s}\neq D_{t}$ and $T_{s}=T_{s}$. Additionally, some unlabeled target domain data must be available at training time.*

Transductive transfer learning is categorized into two cases: (1) source and target adopt different feature domains $X_{s}\neq X_{t}$; (2) source and target adopt the same feature domains, but the probability distributions of their observations are different $P\left(X_{s}\right)\neq P\left(X_{t}\right)$. This case is referred to as domain adaptation.

Transfer learning for cross-domain variations in the context of sensor-based monitoring can be categorized into cross-user, cross-modality, cross-platform, and cross-location activity recognition [13]. Researchers have proposed several transfer learning techniques to address the challenge of domain shift in the context of sensor-based systems. Prior research utilized intra-affinity of classes to perform intra-class knowledge transfer where 61.4% accuracy for cross-location and cross-subject transfer learning was achieved [14]. Another study proposed a feature-level transfer learning approach for activity recognition where 93.1% accuracy for cross-subject knowledge transfer was obtained [13]. Prior research also developed OptiMapper as a transfer learning framework for the case where the target domain provides data about only a subset of the classes [15]. However, as the degree of divergence between source and target domains grows, the transfer learning task becomes more challenging. These gaps result in a performance decline of pre-trained activity recognition algorithms. ActiLabel is proposed as a combinatorial optimization to address the problem of autonomous learning across highly diverse domains (e.g., across different sensor modalities, sensor locations, or users).

Prior research also proposed a deep convolution recurrent neural network to automate the process of feature extraction and to capture general patterns from activity data [16]. In deep learning based methods, the goal is to have a pre-trained model obtained in a source domain and make it fit to our learning problem in the target domain by adding one more training step. Additionally, deep learning based methods need a labeled set for training and do not aim to label the unlabeled samples in the target domain. However, ActiLable is model-agnostic and does not rely on a specific type of machine learning model. We create a labeled training dataset in the target domain by mapping the target sensor data onto the labeled samples in the source domain prior. This model-agnostic approach allows designers to utilize the obtained training dataset and develop the machine learning of their choice for use in a target domain without being limited to the specific architecture that exists in the source domain.

We also note that deep learning models may perform very poorly in profoundly different domains such as cross-modality knowledge transfer or when the two domains exhibit a substantial amount of shift in the distribution of the sensor data. For example, previous research achieved only 54.2% accuracy in classifying human gestures using deep learning with computationally dense algorithms when the system was used with sensors of different modalities than that of training [8,17]. More advanced models combine knowledge of transfer and deep learning [18]. There have been studies attempting to transfer different layers of deep neural networks across different domains. In one study, a cross-domain deep transfer learning method was introduced that achieved 64.6% accuracy with four activity classes for cross-location and cross-subject knowledge transfer [9]. Unlike our transductive transfer learning approach in this paper, these approaches fall within the category of inductive transfer learning, where some labeled instances are required in the target domain.

### 1.2. Graph Modeling

Many areas of machine learning, such as clustering/community detection, dimensionality reduction, and semi-supervised learning, employ neighbor graphs to extract high-level global structures from local information within a dataset [19,20]. As an example, nearest neighbor graphs are commonly used to classify unknown events using feature representations. During the classification process, certain features are extracted from unknown events and classified based on the features extracted from their k-nearest neighbors.

The nearest neighbor or, in general, the k-nearest neighbor (k-NN) graph of a dataset is obtained by connecting each data point to its *k* closest points from the dataset. The closeness is defined based on a distance metric between the data points. The symmetric k-NN graphs are a special case where each point is connected to another only if both are in the *k* nearest vicinity of each other.

**Definition** **3** (Symmetric k-NN Grpah). *A symmetric k-NN graph is a directed graph G=(V,E), where V is the set of vertices (i.e., data observations) and E is the set of edges. $V_{i}$ is connected to vertex $V_{j}$ if $V_{j}$ is one of the k-NNs of $V_{i}$ and vice versa according to a distance function δ:V×V→R.*

Community detection algorithms are widely used to identify clusters in large-scale network graphs. Clusters, which represent groups of densely connected vertices with sparse connections to each other, often provide useful structural information [21]. Recent research compared different community detection algorithms with clustering techniques suggesting that detecting communities from a network representation of data could result in better clustering performance compared to traditional clustering algorithms [22,23]. We define some of the essential features related to community detection in network graphs in the following.

**Definition** **4** (Cut). *Given a graph G($V_{N}$,$E_{N}$) and communities C = {$C_{1}$, ⋯, $C_{K}$}, “Cut” between communities $C_{i}$ and $C_{j}$ is defined as the number of edges (u,v) with one end in $C_{i}$ and the other end in $C_{j}$, that is,*

(1) $$Cut\left(C_{i},C_{j}\right)=\left|\left(u,v\right)\in E_{N}:u\in C_{i}\;\;\&\;\;v\in C_{j}\right|$$

**Definition** **5** (Cluster Density). *Given a graph G($V_{N}$,$E_{N}$) and communities C = {$C_{1}$, ⋯, $C_{K}$} within the graph G, “community density”,* Δ*($C_{i}$), for community $C_{i}$ is defined as the number of edges (u,v) with both ends residing in $C_{i}$.*

(2) $$\Delta \left(C_{i}\right)=\left|\left(u,v\right)\in E_{N}:u\in C_{i}\;\;\&\;\;v\in C_{i}\right|$$

**Definition** **6** (Community Size). *Given a graph G($V_{N}$,$E_{N}$) and communities C = {$C_{1}$, ⋯, $C_{K}$} within the graph G, “Community Size”, σ($C_{i}$), for community $C_{i}$ is defined as the number of vertices that reside in $C_{i}$.*

(3) $$\sigma \left(C_{i}\right)=\left|v\in V_{N}:v\in C_{i}\right|$$

## 2. Problem Statement

**Figure 1:**
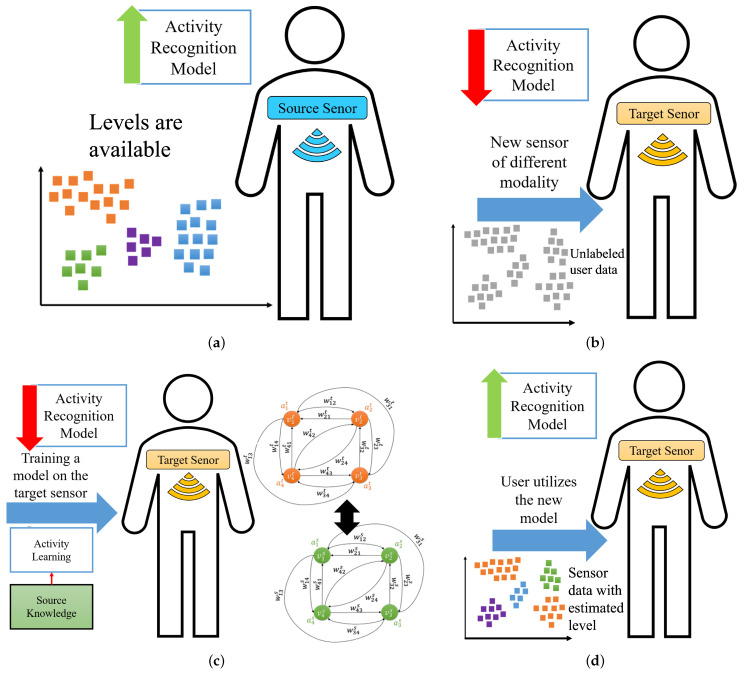
Deployment of ActiLabel in real-world environments.

Figure 1 depicts an activity recognition framework when it is adopted on a new wearable sensor of different modality from the initial one. As shown in Figure 1a, an activity recognition system consisting of a wearable sensor (e.g., accelerometer) uses a model learned based on annotated data. We refer to this setting as source domain. As shown in Figure 1b, when the user replaces the existing sensor with a new sensor with different modality (e.g., stretch sensor), the performance of the existing model declines. We refer to this setting as the target domain. To overcome this challenge, we need to label the dataset autonomously in the new setting (e.g., new sensor modality), as shown in Figure 1c. Finally, as shown in Figure 1d, a more accurate classifier is trained using the labeled training data in the target domain.

### 2.1. Problem Definition

We represent each sensor observation in an arbitrary domain (e.g., target domain) as a k-dimensional feature vector $X_{i}=\left\{f_{i1},f_{i2},\ldots ,f_{ik}\right\}$, which are computed from a given time window. We define the activity recognition task as assigning activity label $l_{i}$ to an observation $X_{i}$ given a set of possible labels $L=\{l_{1},l_{2},\ldots ,l_{m}\}$. The problem is to create a labeled dataset in the target domain by transferring the knowledge from the labeled observations in the source domain such that the activity misclassification in the target is minimized. We define this problem as combinatorial transfer learning.

**Problem** **1** (Combinatorial Transfer Learning (CTL)). *Let X = {$X_{1}$, $X_{2}$, ⋯, $X_{n}$} be the set of sensor observations (i.e., sensor readings represented in feature space) captured in the target domain. Furthermore, let L = {$l_{1}$, $l_{2}$, ⋯, $l_{m}$} be the set of activity labels in the source domain that the target domain aims to detect. Combinatorial transfer learning involves assigning labels to $X_{i}$ and developing a classification model using the labeled data such that the classification error is minimized.*

Because mislabeled sensor data adversely impacts the performance of the learned classifier, CTL can be viewed as the problem of assigning labels $l_{j}\in L$ to target observations $X_{i}$ in X such that the error of label assignment is minimized.

### 2.2. Problem Formulation

We formulate the CTL described in Problem 1 as follows:

(4) $$Minimize\;\;\;\sum_{i=1}^{n}\epsilon_{ij}x_{ij}$$

Subject to:

(5) $$\;\sum_{i=1}^{n}x_{ij}\leq \lambda_{j}\;\;\;\;\;\;\forall j\in \left\{1,\cdots ,m\right\}$$

(6) $$\;\sum_{j=1}^{m}x_{ij}=1,\;\;\;\;\;\;\forall i\in \left\{1,\cdots ,n\right\}$$

(7) $$\;x_{ij}\in \left\{0,1\right\}$$

where $x_{ij}$ is a decision variable indicating whether or not $X_{i}$ is assigned label $l_{j}$, and $\epsilon_{ij}$ denotes error due to such a labeling. The constraint in (5) guarantees that at most $\lambda_{j}$ target observations are assigned label $l_{j}$. Without such a constraint, a trivial solution is to label no observations in the target domain. The constraint in (6) ensures that only one label is assigned to each observation $X_{i}$ in the target domain.

### 2.3. Solution Overview

The difficulty in solving Problem 1 arises not only from the hardness of the problem but also from the fact that parameters $\lambda_{j}$ and $\epsilon_{ij}$ are not known *a priori*. Therefore, the solution to the CTL problem in (4)–(7) needs to estimate $\lambda_{j}$ and $\epsilon_{ij}$ first. Since assigning a label to every observation in the target is unlikely to result in a high labeling accuracy, we propose to find groups of similar target observations that are reliable to receive the same label. Unsupervised clustering is one approach to divide observations into groups, exclude noisy observations from the labeling process, and therefore increase the specificity of the labeling. We can estimate the value of λ by identifying clusters of observations that are safe to receive the same activity label, namely, core clusters. Let $C_{i}^{D}=\left\{X_{1},X_{2},\ldots ,X_{k}\right\}$ be a $i^{th}$ cluster in domain *D*. After clustering the target data, the goal is to assign activity labels to the core clusters such that the label misassignment is minimized. Therefore, the CTL problem can be reformulated as below.

(8) $$Minimize\;\;\;\sum_{i=1}^{n}\sum_{j=1}^{m}\alpha_{ij}\epsilon_{ij}$$

Subject to:

(9) $$\;\sum_{j=1}^{m}\alpha_{ij}=1,\;\;\;\;\;\;\forall i\in \left\{1,\cdots ,n\right\}$$

where $\alpha_{ij}$ is a binary variable indicating whether or not $i^{th}$ cluster in the target is assigned with label $l_{j}$ from $j^{th}$ cluster in the source domain, and $\epsilon_{ij}$ denotes the assignment error. $\epsilon_{ij}$ can be estimated as a structural dissimilarity between cluster $C_{i}^{t}$ in the target and cluster $C_{j}^{s}$ in the source domain. Cluster $C_{j}^{s}$ is a cluster of observations with label $L_{j}$ in the source domain. Note that computing the dissimilarity between the clusters will be further discussed in the next steps. The constraint in Equation (9) ensures that only one label is assigned to each core cluster $c_{i}$ from the target domain.

(10) $$\;\alpha_{ij}=\left\{\begin{array}{cc} 1, & \mathrm{if}\;\mathrm{label}\;l_{j}\;\mathrm{is}\;\mathrm{assigned}\;\mathrm{to}\;\mathrm{cluster}\;C_{i}\\ 0, & \mathrm{otherwise} \end{array}\right.$$

## 3. Actilabel

**Figure 2:**
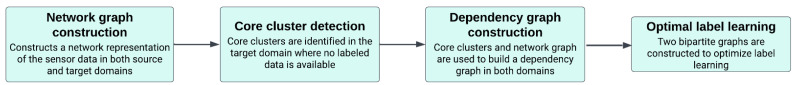
*ActiLabel* comprises of several steps. *Network graph construction* is done by quantifying the pairwise similarity of sensor observations using statistical features and semantic information; *Core clusters* are directly obtained through the available class labels; *Dependency graph* captures the structural relationships between activity classes; and *Optimallabel learning* uses two bipartite, one of which captures the cost of mapping each vertex in the source dependency graph to every vertex in the target dependency graph. The other one quantifies the costs of edge-wise mapping between the two domains.

**Figure 3:**
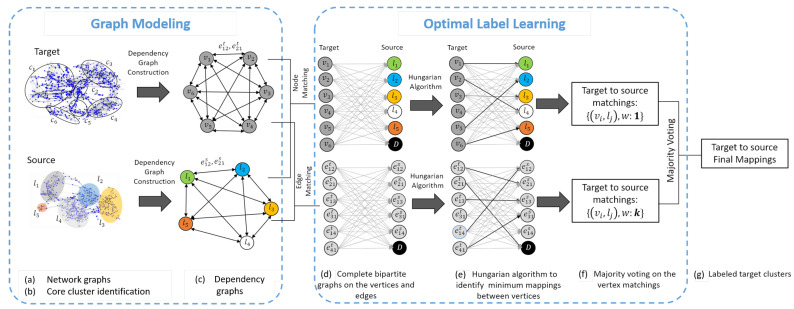
An overview of ActiLabel design including graph modeling and optimal label learning.

We propose ActiLabel as a solution to Equation (8). The overall approach in ActiLabel is illustrated in Figure 2 and Figure 3. The design process in ActiLabel involves the following steps, where we refer to the first two steps as *graph modeling* and the next two steps as *optimallabel learning*.

1. *Network graph construction:* we first construct a network representation of sensor readings and quantify the pairwise similarity of the network nodes (i.e., sensor observations) using a combination of statistical features and semantic information about the network Figure 3a.
2. *Core cluster identification:* we use the network graph to identify core clusters in the target domain where no labeled data are available. For the source domain, the core clusters/classes are directly obtained through the available class labels as shown in Figure 3b.
3. *Dependency graph construction:* we use the core clusters and network graph to build a dependency graph in both domains, taking into account inter-class similarities as shown in Figure 3c.
4. *Optimal Label Learning*: we use the dependency graphs of the source and target domains to build two bipartite graphs. The first bipartite graph captures the cost of mapping each vertex in the source dependency graph to every vertex in the target dependency graph. The second bipartite graph quantifies the costs of edge-wise mapping between the two domains, as shown in Figure 3d–f.

The process of ActiLabel is summarized in Algorithm 1.

**Algorithm 1** ActiLabel    **Input:**
* $D_{t}$, unlabeled target dataset, {$D_{s}$, $L_{s}$}, labeled source dataset.*
    **Result:** *Labeled target dataset, {$D_{t}$, $L_{t}$}*    **Graph Modeling:**                    ▹ (Section 3.1)1:        *Construct network graphs in both domains;*          ▹ (Section 3.1.1)2:        *Identify core clusters in both domains;*               ▹ (Section 3.1.2)3:        *Build dependency graphs;*                  ▹ (Section 3.1.3)4:        *Extract structural relationships among the core clusters in both domains;*     **Optimal Label Learning**                ▹ (Section 3.2)5:        *Perform graph-level min-cost mapping from target to source;*6:        *Assign labels to the observations in target;*7:        *Train activity recognition model in target using new labels;*


### 3.1. Graph Modeling

**Figure 4:**
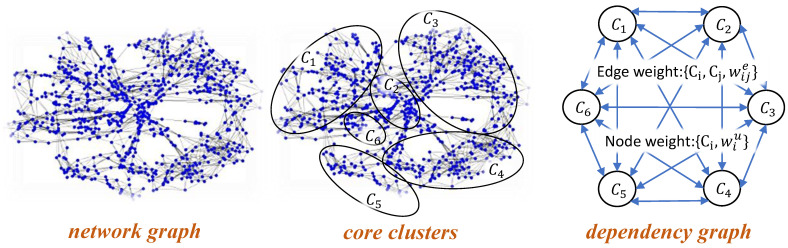
Graph modeling phases.

The goal of our graph modeling is to construct a dependency graph that captures structural dependencies among the events (i.e., physical activities) in both target and source domains. Such dependency graphs are then used in *optimal label learning* to label sensor observations and generate a training dataset in the target domain. As shown in Figure 4, our graph modeling consists of three phases: (i) network graph construction; (ii) core cluster identification; and (iii) dependency graph construction. This section elaborates on each phase.

#### 3.1.1. Network Graph Construction

We initially build a network representation of the sensor observations to quantify the amount of similarity between pairs of observations. To this end, we construct a symmetric k-nearest-neighbor network on the sensor data. The symmetric property of the network graph eliminates many edges from inclusion in the network, thereby reducing the complexity of future computations in ActiLabel.

**Definition** **7** (Network Graph). *The network graph refers $G_{N}$($V_{N}$,$E_{N}$) is a symmetric k-NN graph where vertices are a feature representation of the sensor data and distance function δ computes the cosine similarity between the features.*

Given the high dimensional feature space, we use Cosine distance as the measure of affinity between each pair of sensor observations $X_{i}$ and $X_{j}$, and as the distance function $\delta (v_{i},v_{j})$ used to construct the network graph.

(11) $$\delta \left(v_{i},v_{j}\right)=cos\left(X_{i},X_{j}\right)=\frac{X_{i}\cdot X_{j}}{\left|\right|X_{i}\left|\right|\cdot \left|\right|X_{j}\left|\right|}$$

#### 3.1.2. Core Cluster Identification

To identify core clusters in ActiLabel, we propose a graph-based clustering algorithm similar to the approach in prior research [24]. We refer to this approach as *core cluster identification* (CCID). The core cluster identification algorithm is applied to the network graph *G*($V_{N}$,$E_{N}$). We first partition the network graph into multiple communities of approximately the same vertex size using a greedy community detection technique. We then merge communities with the highest similarity score based on their dendrogram structure.

The amount of similarity $\alpha_{i,j}$ between communities $C_{i}$ and $C_{j}$ is measured as the ratio of the number of edges between the two communities (i.e., Cut($C_{i}$,$C_{j}$)) to the average number of edges that reside within the two communities. Therefore, the similarity score of $\alpha_{i,j}$ is given by

(12) $$\alpha \left(i,j\right)=\frac{Cut(C_{i},C_{j})}{\frac{|C_{i}\left|+\right|C_{j}|}{2}}$$

where the terms $|C_{i}|$ and $|C_{j}|$ denote the number of edges that reside in $C_{i}$ and $C_{j}$, respectively. Note that the similarity score α is defined such that it is not adversely influenced by the size of communities in unbalanced datasets.

#### 3.1.3. Dependency Graph Construction

To capture high-level structural relationships among sensor observations, we devise a structural dependency graph where the core clusters identified previously represent vertices of the dependency graph.

**Definition** **8** (Dependency Graph). *Given a network graph G($V_{N}$,$E_{N}$) where $|V_{N}|$ = |X| and core clusters C = {$C_{1}$, ⋯, $C_{K}$} obtained from the network graph, we define dependency graph G($V_{D}$, $E_{D}$, $W_{D}^{v}$, and $W_{D}^{e}$) as a weighted directed complete graph as follows. Each vertex $u_{i}\;inV_{D}$ is associated with a core cluster $C_{i}\in C$. Thus, $|V_{D}|$ = |C|. Each vertex $u_{i}\in V_{D}$ is assigned a weight $w_{i}^{u}$ given by*

(13) $$w_{i}^{u}=\frac{\Delta (C_{i})}{\sigma (C_{i})}$$

*where $\Delta (C_{i})$ and $\sigma (C_{i})$ refer to cluster density and cluster size, respectively, for core cluster $C_{i}$. Each edge $\left(u_{i},u_{j}\right)\in E_{D}$, associated with core clusters $C_{i}$ and $C_{j}$, is assigned a weight $w_{ij}^{e}$ given by*

(14) $$w_{ij}^{e}=\frac{Cut(C_{i},C_{j})}{\sigma (C_{j})}$$

### 3.2. Optimal Label Learning

Algorithm 2 summarizes the steps for optimal label learning. The goal of the optimal label learning is to find a mapping from the dependency graph in the target domain to that of the source domain. We note that graph isomorphism algorithms are not applicable to our graph-level mapping problem because graph isomorphism algorithms only consider the structure of the graphs and do not take into account important information such as edge weights and vertex weights in our dependency graphs [25]. The core of our optimization in label learning is graph-level mapping, where we aim to find a mapping from the dependency graph in the target domain to that of the source domain while minimizing the amount of mapping error. We refer to this optimization problem as min-cost dependency graph mapping and define it as follows.

**Algorithm 2** Optimal Label Learning
     **Input:** 
*$G_{D}^{t}$ and $G_{D}^{s}$, dependency graphs for target and source domains.*     **Result:** 
*Labeled target dataset, {$D_{t}$, $L_{t}$}*1:*  Construct bipartite graph $BG_{e}$ using edge components;*2:*  Obtain bipartite mapping $M_{e}$ on $GB_{e}$;*3:*  Construct bipartite graph $BG_{v}$ on vertex components;*4:*  Obtain bipartite mapping $M_{v}$ on $GB_{v}$;*5:*  Construct bipartite graph $BG_{c}$ using $M_{e}$ and $M_{v}$;*6:*  Obtain bipartite mapping OptMapping on $GB_{c}$;*7:*  Assign source labels to appropriate core clusters in target using OptMapping;*

**Problem** **2** (Min-Cost Dependency Graph Mapping). *Let $G_{D}^{s}$ and $G_{D}^{t}$ denote dependency graphs obtained from datasets in the source and target domains, respectively. The min-cost dependency graph mapping is to find a mapping $R:G_{D}^{t}\to G_{D}^{s}$ from $G_{D}^{t}$ to $G_{D}^{s}$ such that the cost of such mapping is minimized.*

Problem 2 can be viewed as a combinatorial optimization problem that finds an optimal mapping in a two-tier fashion: (i) it initially performs component-level mappings where vertex-wise and edge-wise mappings are found between source and target dependency graphs; and (ii) it then uses the component-level mappings to reach a consensus about the optimal mapping for the problem as a whole. Such a two-level mapping problem can be represented using the objective in (15).

(15) $$\;Minimize\;\;\;\sum_{i=1}^{|V_{D}^{t}|}\sum_{j=1}^{|V_{D}^{s}|}1-\frac{\mu (i,j)}{M}$$

where μ(i,j) represents the number of mappings between $v_{i}\in V_{D}^{t}$ and $v_{j}\in V_{D}^{s}$ obtained through the component-level optimization. Furthermore, *M* is a normalization factor that is equal to the total number of component-wise mappings. The objective in (15) attempts to minimize the amount of mapping costs at the graph-level and, therefore, can be viewed as the objective for Problem 2.

**Figure 5:**
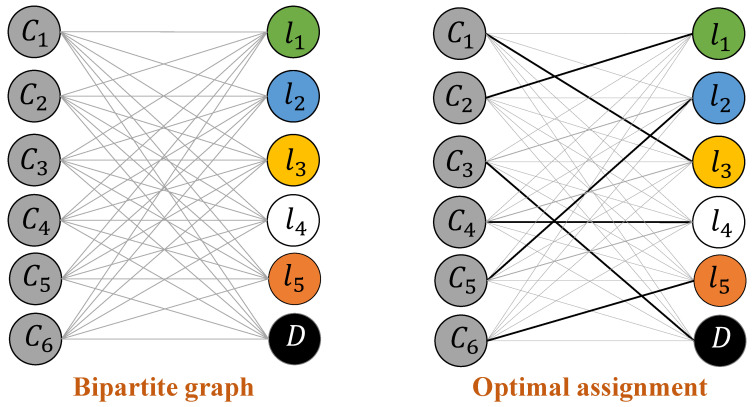
Optimal label assignment includes constructing a component-wise bipartite graph and finding an optimal mapping of those components from target to source.

We build a weighted complete bipartite graph on the elements of the similarity matrix to find the minimum double-cost mapping. Figure 5 is an illustration of such a bipartite graph, where the nodes on the left shore of the graph represent elements (e.g., cluster density) of the target similarity matrix and the nodes on the right shore of the bipartite graph are associated with corresponding elements (e.g., cluster density) in the source similarity matrix.

In constructing a bipartite graph, a weight $\omega_{ij}$ is assigned to the edge that connects node *i* in the target side to nodes *j* in the source side. This weight also represents the actual mapping cost and is given by

(16) $$\omega_{ij}=\left|w_{si}-wtj\right|$$

where $w_{si}$ and wtj are, respectively, the weight values associated with element *i* in the source domain and component *j* in the target domain. We note that these weights can be computed using (13) and (14) for vertex-wise mapping and edge-wise mapping, respectively. We also note that if the number of components in the source and target were not equal, we could add dummy nodes to one shore of the bipartite graph to create a complete and balanced bipartite graph.

We use the Hungarian algorithm (a widely used weighted bipartite matching algorithm with $O(m^{3})$ time complexity) [26] to identify an optimal mapping from the nodes on the left shore of the bipartite graph to the nodes on the right shore of the graph.

The last step is to assign the labels mapped to each cluster to the target observations within that cluster. A classification model is trained on the labeled target dataset for physical activity recognition.

## 4. Time Complexity Analysis

**Lemma** **1.** *The optimal label learning phase in ActiLabel has a time complexity of $O(n+m^{3})$, where n denotes the number of sensor observations and m represents the number of classes.*

**Proof.**  To learn the optimal labels, ActiLabel finds an optimal matching between source and target dependency graphs given the node and edge weight values. We solve the dependency graph matching problem by running the Hungarian algorithm three times. Given that the number of the core clusters is proportional to the number of labels, *m*, the time complexity of running Hungarian algorithm three times is $O(m^{3})$. Distributing the labels to the cluster members can be done in O(n). Therefore, the optimal label learning phase has a time complexity of $O(n+m^{3})$. □

The last step is to assign the labels to the target observations within each cluster. A classification model is trained on the labeled target dataset for physical activity recognition.

**Theorem** **1.** *The time complexity of ActiLabel is quadratic in the number of sensor observations, n.*

**Proof.**  Assuming that the number of classes, *m*, is much smaller than the number of sensor observations, *n*, (i.e., m≪n), the proof follows Lemma 2 and Lemma 1. □

**Theorem** **2.** *CTL is NP-hard.*

**Proof.**  Proof by reduction is done from the well-known generalized assignment problem [27].

Theorem 2 claims that the CTL problem discussed in Problem 1 and formulated in (4)–(7) is NP-hard. In this section, we prove that Problem 1 is NP-hard by reduction from the generalized assignment problem (GAP), which is known to be NP-hard [27]. The generalized assignment problem aims to assign a set of tasks to a set of agents while minimizing the total assignment cost. It needs to guarantee that each task is assigned to one and only one agent. In GAP, each agent has a limited capacity. Additionally, each task requires a given number of resources of each agent. Each task needs to be assigned to only one agent.

An instance of GAP is given by (*I*,*J*,*A*,*B*,*C*) where *I* = {1, 2, ⋯, *n*} represents the set of *n* tasks; *J* = {1, 2, ⋯, *m*} denotes the set of *m* agents; *B*={$b_{1}$, $b_{2}$, ⋯, $b_{m}$} maintains resource capacity $b_{j}$ for each agent *j* in *J*; *A* = {$a_{ij}$} represents resource $a_{ij}$ needed if task *i* is assigned to agent *j*; and finally *C*={$c_{ij}$} represents the cost of assigning task *i* to agent *j*. The generalized assignment problem can be formulated as follows:

(17) $$Minimize\;\;\;\sum_{i=1}^{n}\sum_{j=1}^{m}c_{ij}x_{ij}$$

Subject to:

(18) $$\sum_{i=1}^{n}a_{ij}x_{ij}\leq b_{j}\;\;\;\;\;\;\forall j\in \left\{1,\cdots ,m\right\}$$

(19) $$\sum_{j=1}^{m}x_{ij}=1\;\;\;\;\;\;\forall i\in \left\{1,\cdots ,n\right\}$$

(20) $$x_{ij}\in \left\{0,1\right\}$$

where $x_{ij}$ is a decision variable indicating whether or not task *i* is assigned to agent *j*.

Consider an instance of the generalized assignment problem, (*I*,*J*,*A*,*B*,*C*). This problem can be reduced to the combinatorial transfer. In fact, the generalized assignment problem is equivalent to the CTL with

(21) J=X

(22) I=L

(23) $$a_{ij}=1\;\;\;\;\;\;\;\;\;\;\;\;\forall i,j$$

(24) $$b_{j}=\lambda_{j}\;\;\;\;\;\;\;\;\;\;\;\;\forall j$$

(25) $$c_{ij}=\epsilon_{ij}\;\;\;\;\;\;\;\;\;\;\;\;\forall i,j$$

□

**Lemma** **2.** *The graph modeling in ActiLabel has a time complexity of $O(n^{2})$, where ‘n’ denotes the number of sensor observations.*

**Proof.**  Lemma 2 claims that the complexity of the graph modeling phase in ActiLabel is $O(n^{2})$, where ‘*n*’ represents the number of sensor observations. Here, we provide the proof for this claim.

The graph modeling phase includes three steps: network graph construction, core cluster identification, and dependency graph construction, which have a complexity of $O(n^{2})$, $O(nlog^{2}\left(n\right)+m^{3})$, and O(m), respectively, as discussed below.

Our introduced network graph in ActiLabel is a *k*nn graph constructed using the input sensor observations. Constructing a *k*nn graph requires computing pairwise distances between sensor observations. Therefore, the *k*nn construction process has a time complexity of $O(n^{2})$.

The core cluster identification algorithm consists of partitioning the network graph and merging the partitions into a final set of clusters. We use the Clauset–Newman–Moore greedy modularity maximization algorithm for network graph partitioning. Because the network graph is sparse, the partitioning algorithm runs in $O(nlog^{2}\left(n\right))$ [28]. In the following, we show that the cluster merging process has a time complexity of $O(m^{3}+mn)$. Therefore, assuming n>m, the core cluster identification algorithm has a time complexity of $O(nlog^{2}\left(n\right)+m^{3})$

The cluster merging process requires (i) a computing pair-wise similarity between the clusters in (12); (ii) finding a pair of clusters that are most similar; and (iii) merging the two clusters, which involves updating the membership of the sensor observations that reside in the merged clusters. We note that, in the worst case, steps (ii) and (iii) will repeat until the entire network graph is merged into a single cluster. To compute pair-wise cluster similarity, we use a fast algorithm that goes over non-zero elements of the adjacency matrix (e.g., edges in the network graph) only once. For each non-zero element, if the adjacent vertices in the network graph belong to the same cluster, we update the cluster weight; otherwise, we update the edge weight between the two clusters based on the similarity values. Therefore, computing the similarity measures runs in O(n). Note that because the network graph is sparse, |E|∼|V|=n. Because the number of clusters is proportional to the number of labels, *m*, the number of cluster-pairs is $O(m^{2})$. Therefore, finding a cluster-pair with maximum similarity takes $O(m^{2})$ to complete. Finally, updating the cluster membership for data points that reside in the merged clusters takes O(n). Note that because steps (ii) and (iii) can repeat for at most *m* times, the complexity of combined steps (ii) and (iii) is $O(m^{3}+mn)$. Combining complexity of the three steps (i), (ii), and (iii) in cluster merging process will give us an overall complexity of $O(m^{3}+mn+n)$ = $O(m^{3}+mn)$.

The dependency graph is a weighted complete graph that is built on the core clusters. The process to compute edge weights and vertex weights in such a graph is similar to computing the pair-wise similarity score while merging the initial clusters. All the edge weights and vertex weights can be therefore calculated during the cluster merging process described earlier. Given that the number of the final clusters is proportional to the number of the labels, *m*, the dependency graph construction can run O(m).

Combining time complexities for network graph construction, core cluster identification, and dependency graph construction will give us $O(n^{2}+nlog^{2}\left(n\right)+m^{3}+m)$ = $\left(n^{2}+m^{3}\right)$. Assuming that in most real applications the number of sensor observations is orders of magnitude larger than the number of class labels, we can conclude that the complexity of the graph modeling phase is ActiLabel is $O(n^{2})$. Hence,

(26) $$\begin{array}{c} O\left(n^{2}+nlog^{2}\left(n\right)+m^{3}+m\right)=O\left(n^{2}+m^{3}\right)=O\left(n^{2}\right) \end{array}$$

□

## 5. Experimental Setup

### 5.1. Datasets

We used three sizeable human activity datasets to evaluate the performance of ActiLabel. We refer to these datasets as PAMAP2, a physical activity monitoring dataset used in [29]; DAS, daily and sport activity dataset used in [30]; and Smartsock, a dataset containing ankle-worn sensor data used in [31]. These datasets contained sensor data with a variety of sensor modalities such as accelerometer, gyroscope, magnetometer, temperature, stretch sensor, and heart rate monitor. They also provided data collected with 29 subjects. The number of wearing sites varied across the datasets, with a total of 8 body locations for the three datasets. Table 1 has provided a summary of the datasets utilized in this study.

### 5.2. Pamap2

**Figure 6:**
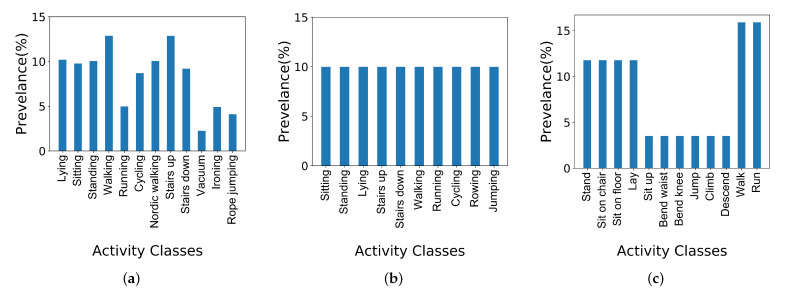
Prevalence of physical activities in the PAMAP2 dataset.

The data in PAMAP2 are collected from 9 participants performing 24 physical activities of daily livings while wearing 3 IMUs (inertial measurement units) on their chest, ankle, and hand while also wearing a heart rate monitoring device on the chest. The IMUs recorded accelerometer (@100 Hz), gyroscope (@100 Hz), orientation (@100 Hz), and temperature (@100 Hz) data, and the heart rate monitor recorded heart rate information (@9 Hz) during the experiments. We only consider 12 activities for our analysis in this paper because there were only 12 activities in the dataset that were performed by all the 15 subjects. As Figure 6a, which visualizes the prevalence of the activities, suggests, PAMAP2 is an imbalanced dataset.

### 5.3. Das

DAS dataset is a collection of 19 sports physical activities performed by eight subjects between the ages of 20 and 30 (four females and four males). The subjects wore the data collection devices, embedding accelerometer (@25 Hz), gyroscope (@25 Hz), and magnetometer (@25 Hz) sensors, on their torso, left arm, right arm, left leg, and right leg. Some of the activities were sitting, standing, lying on the back and right side, ascending and descending stairs, walking, running, cycling, rowing, and jumping. DAS is a balanced dataset as illustrated in Figure 6b.

### 5.4. Smartsock

The Smartsock dataset was collected from 12 participants (four females and eight males) aged between 23 and 31. The participant performed 12 different physical tasks while wearing a Smartsock prototype on the dominant foot that measured the circumference of the ankle using a stretch sensor. They also wore an accelerometer sensor on the chest during the protocols. The activities were sit in chair, sit on floor, lay on floor, bend at knees, bend at waist, jump in place, descending stairs, walking, and running. Figure 6c visualizes the prevalence of the physical activities in the Smartsock dataset. The majority of the observations belonged to the walking and running activities.

### 5.5. Comparison Methods

We compare the performance of ActiLabel with the following algorithms. We deploy the 5-NN classifier on the feature representation of the data as the baseline classifier for the Baseline, DirectMap, and upper-bound, as suggested in the *Results* section.

- *Baseline* refers to the case where we learn a feature-based activity recognition model in the source domain and use it for activity recognition in the target domain.
- *Deep Convolution LSTM (ConvLSTM)* refers to using a deep convolution LSTM model that was learned in the source domain and was utilized for activity recognition in the target domain. The deep ConvLSTM consists of one layer of input, four layers of convolution, two dense layers consisting of LSTM cells as the hidden units, and a softmax layer as the output of the model as proposed by [16].
- *DirectMap* directly maps core clusters in a target domain to activity classes in a source domain using the Hungarian algorithm. This algorithm assigns the labels from the source cluster to the closest cluster in the target domain based on a similarity measure on the mean value of the data points in each cluster.
- *Upper-bound* assumes that the actual labels are available in the target domain.

We assess the performance of ActiLabel and these competing algorithms in three transfer learning scenarios as follows: (i) cross-modality transfer refers to the case when sensors in the two domains have different modalities (e.g., the accelerometer and the gyroscope); (ii) cross-subject refers to transfer learning across two different human subjects; and (iii) cross-location refers to the case when the location of the wearable sensor is different in the target domain from that in the source domain.

### 5.6. Implementation Details

The datasets are divided into 50% training, 25% test, and 25% validation parts with no overlap to avoid possible bias. The input features are extracted from a 2-second window of data. We extracted an exhaustive set of time-domain features from a sliding window of size 2 s with 25% overlap. Table 2 lists the extracted features, which are shown to be useful in human physical activity estimation using inertial sensor data [32,33].

We performed dimensionality reduction based on the UMAP [34] algorithm prior to clustering since distance-based clustering algorithms are negatively affected by high dimensionality in feature space. The *k* parameter in the Baseline graph construction was set to the 2% or 5% of the size of the Baseline graph, as suggested by the results in Section 6.1.

In the following subsections, we discuss performance metrics, comparison algorithms, and parameter settings for our evaluation of ActiLabel.

### 5.7. Evaluation Metrics

We adopt four metrics to evaluate the performance of ActiLabel in this paper.

- To evaluate the performance of the core cluster identification, we report *normalized mutual information (NMI)* and *purity*. NMI is an entropy based method that is a measure of information sharing between the ground truth labels and clustering. Purity shows how much each cluster contains a single class.
  (27) $$NMI\left(L,C\right)=\frac{2\times I(L;C)}{\left[H(L)+H(C)\right]}$$
  where *L* is the actual class labels and *C* is the cluster labels. Function H(.) computes the entropy of the input vector, and I(Y;C) denotes the mutual information between *Y* and *C*. To calculate purity, we assume each cluster $C_{i}$ is assigned to the most frequent label label in the cluster.
  (28) $$purity\left(C,L\right)=\frac{\sum_{k}\underset{j}{max}\left|w_{k}⋂c_{j}\right|}{N}$$
  where $C=\{c_{1},c_{2},\ldots ,c_{k}\}$ is the set of clusters and *L* is the set of labels. Both NMI and purity are normalized between 0 and 1 [35]
- To evaluate the performance of the double-weighted matching algorithm, we report *labeling accuracy*. The labeling accuracy is defined as the ratio of the target sensor observations that are correctly mapped to an activity label in the source.
  (29) $$Labeling-Accuracy=\frac{\sum_{i=1}^{k}\frac{{TP}_{i}+{TN}_{i}}{{TP}_{i}+{TN}_{i}+{FP}_{i}+{FN}_{i}}}{k}$$
  where *k* refers to the number of classes. For each cluster $c_{i}$ with label $l_{i}$, ${TP}_{i}$ refers to the samples that are correctly labeled as $l_{i}$, ${FP}_{i}$ represents the samples that are falsely labeled as $l_{i}$, ${TN}_{i}$ is defined as the samples that are correctly not labeled as $l_{i}$, and ${FN}_{i}$ represents the samples that are falsely not labeled as $l_{i}$
- To evaluate the performance of the ActiLabel framework as a whole, we report the *F*1*-score* of the activity recognition algorithm that is autonomously trained because it better represents the performance of the model when dealing with imbalanced data [36]. F1-score is defined as the weighted average of the precision and recall [36].
  (30) $$F1-score=\frac{2\times (Recall\times Precision)}{Recall+Precision}$$
  where precision refers to the average agreement of the actual class labels and classifier-predicted labels, and recall is the average effectiveness of the classifier to identify each class label. Precision and recall are computed by the following equations:

(31) $$Precision=\frac{\sum_{i=1}^{k}\frac{{TP}_{i}}{{TP}_{i}+{FP}_{i}}}{k},Recall=\frac{\sum_{i=1}^{k}\frac{{TP}_{i}}{{TP}_{i}+{FN}_{i}}}{k}$$

where *k* refers to the number of classes. For each activity class $A_{i}$ with label $l_{i}$, ${TP}_{i}$ refers to the samples that are correctly classified as $l_{i}$, ${FP}_{i}$ represents the samples that are falsely classified as $l_{i}$, ${TN}_{i}$ is defined as the samples that are correctly not classified as $l_{i}$, and ${FN}_{i}$ represents the samples that are falsely not classified as $l_{i}$ [37].

## 6. Results

As mentioned previously, the main focus of ActiLabel is to create a labeled dataset in a target domain. This dataset can then be used to train an activity recognition model. Therefore, the methodologies presented in this paper are independent of the choice of the classifier that can be used for activity recognition. For validation purposes, however, we performed an extensive experiment to identify the most accurate classification model that can be used for activity recognition. Table 3 compares the F1-score for k-NN with k=5, support vector machine (SVM) with RBF kernel, logistic regression (LR), random forest (RF) with bagging of 100 decision trees, artificial neural network (ANN), Naive Bayes (NB), and quadratic discriminant analysis (QDA). k-NN (K = 5) achieves the highest performance, such as 93.8% average F1-score over different sensor locations in PAMAP2 dataset, 94.5% over different sensor modalities, and 97.1% over different sensor modalities for DAS dataset. ANN achieved the best F1-score for the rest of the cases.

In what follows, we discuss the performance of ActiLabel for core cluster identification, labeling accuracy, and activity recognition accuracy.

### 6.1. Performance of Core Cluster Identification

**Figure 7:**
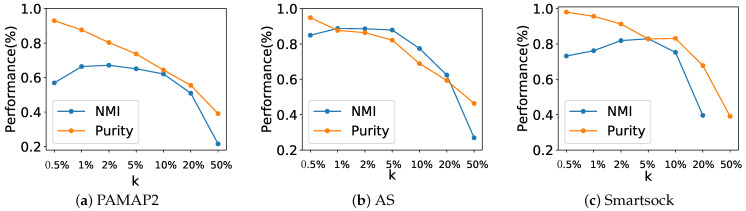
Performance (i.e., normalized mutual information and purity) of core cluster identification versus parameter *k* in network graph construction.

We analyzed the effect of parameter *k* in the *k*-NN network graph on the performance of the core cluster identification as measured by normalized mutual information (NMI) and clustering purity. As shown in Figure 7, the value of parameter *k* is set according to the size of the network graph. Specifically, measure NMI and purity for *k* range from 0.5% to 50% of the network graph size. Note that purity decreases as *k* grows because a higher purity (e.g., 0.85 to 0.98) can be achieved when detecting more clusters. A smaller *k* results in sparser network graph, which in turn leads to the acquisition of more clusters. As shown in Figure 7, NMI achieved its highest value (i.e., 0.67 for PAMAP2, 0.88 for DAS, and 0.83 for Smartsock) when *k* was set to 2% or 5% of the graph network size. This translates into a *k* = 8 for PAMAP2 and Smartsock and k=11 for DAS datasets.

**Figure 8:**
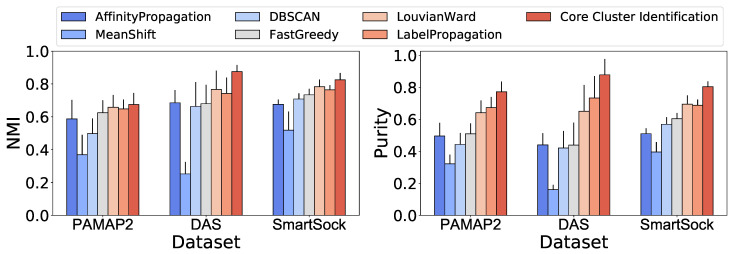
Performance comparison between core cluster identification in ActiLabel and standard clustering and communication detection algorithms.

Figure 8 compares the average NMI score and purity of clustering between the proposed core cluster identification (CCI) method and well-known clustering and community detection algorithms. We chose the algorithms that do not require prior knowledge on the cluster counts because the activity labels are unknown in the target domain. Note that the community detection algorithms were applied to a symmetric k-NN graph (k = 10) built on the feature representation of observation after dimensionality reduction using the UMAP [34] algorithm.

- Affinity propagation is a graph-based clustering algorithm that extracts the clusters by relaying messages between pairs of samples until convergence [38].
- Mean shift is a centroid-based algorithm that extracts clusters on a smooth density of data [39]
- DBSCAN clustering algorithm detects the cluster based on a density measure [40].
- Fast greedy finds the communities in the graph using Clauset–Newman–Moore greedy modularity maximization [28].
- Lovain–Ward detects the communities in the graph by maximizing the modularity using the Louvain heuristics [41].
- Label propagation finds the communities in the graph using a semi-synchronous label propagation method [42].

As shown in Figure 8, CCI outperforms state-of-the-art clustering and community detection algorithms. The NMI for the competing methods ranged from 0.37–0.65 for PAMAP2, 0.25–0.77 for DAS, and 0.52–0.76 for Smartsock. The proposed algorithm CCI increased NMI to 0.67, 0.87, and 0.85 for PAMAP2, DAS, and Smartsock datasets, respectively.

Affinity propagation, DBSCAN, Lovain–Ward, fast greedy, and label propagation algorithms achieved 0.50–0.67, 0.44–0.73, and 0.51–0.69 purity for PAMAP2, DAS, and Smartsock datasets, respectively. Mean shift achieved the lowest purity compared to other comparison algorithms (0.32 for PAMAP2, 0.16 for DAS, and 0.40 for Smartsock). Using our core cluster identification The purity measure reaches 0.77 for PAMAP2, 0.88 for DAS, and 0.80 for Smartsock dataset. Note that the clustering was generally more accurate for Smartsock and DAS datasets because PAMAP2 contained data from sensor modalities (e.g., temperature) that might not be a good representative of the activities of interest.

### 6.2. Labeling Accuracy in ActiLabel

**Figure 9:**
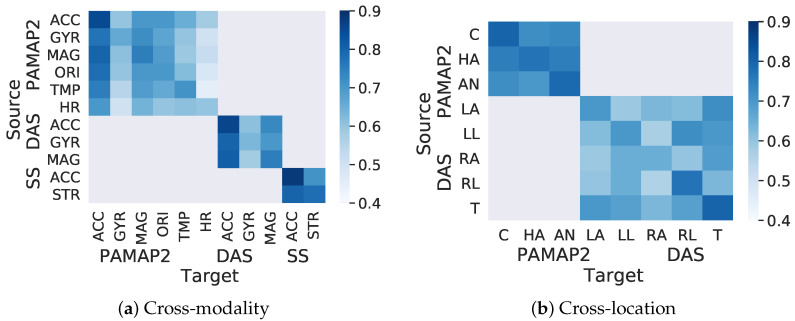
Labeling accuracy of ActiLabel for (**a**) cross-modality and, (**b**) cross-location learning.

Because ActiLabel generates a labeled training dataset in the target domain, it is reasonable to assess the accuracy of the labeling task. Figure 9 shows the labeling accuracy for various transfer learning scenarios and datasets. For brevity, the results from cross-subject labeling are not included in this figure.

#### 6.2.1. Cross-Modality Transfer

As the heatmap in Figure 9a shows, ActiLabel achieved 70.2–88.0% labeling accuracy when the accelerometer was the target modality. With the accelerometer being the target modality, the highest labeling accuracy (>80%) was obtained when the source modality was the magnetometer, the stretch sensor, or another accelerometer. We also observed that the labeling accuracy ranged from 60% to 75% when the target modality was magnetometer or orientation sensor. We also noted that transferring labels between orientation and heart rate sensors achieved the lowest accuracy (i.e., 45–0.65%), mainly because these sensor modalities are not as good representative of the physical activities as the accelerometer. The proposed mapping algorithm obtained >80% labeling accuracy for the remaining transfer scenarios except for “magnetometer to orientation” mapping (77.9%) and for “temperature to temperature” mapping (74.0%).

#### 6.2.2. Cross-Location Transfer

The heatmap in Figure 9b shows the labeling accuracy between sensor locations in PAMAP2 and DAS datasets. Note that the Smartsock dataset contained only one sensor location, and therefore a cross-location transfer did not apply to this dataset. As expected, mapping labels between the same or similar body locations such as “chest to chest”, “hand to hand”, “ankle to ankle”, “torso to torso”, “left arm to left arm”, “left leg to left leg”, and “left arm to right leg” achieved a relatively high labeling accuracy (i.e., >70.3%). Furthermore, ActiLabel achieved 70.3–80.1% labeling accuracy for transfer tasks between chest, ankle, and hand in PAMAP2. One reason for a relatively high labeling accuracy in such transfer tasks involving dissimilar sensor locations is that PAMAP2 contains a rich collection of sensors (accelerometer, gyroscope, magnetometer, orientation, temperature, and heart rate sensors) that provide sufficient information about inter-event structural similarities captured by our label learning algorithms in ActiLabel.

### 6.3. Performance of Activity Recognition

Table 4 shows activity recognition performance (e.g.,F1-score) for ActiLabel as well as the algorithms under comparison, including baseline (BL), deep convolution LSTM (CL), DirectMap (DM), and upper-bound (UB), as discussed previously.

#### 6.3.1. Cross-Modality Transfer

For this scenario, we examined transfer learning across these sensor modalities: accelerometer, gyroscope, magnetometer, orientation, temperature, heart rate, and stretch sensor. The cross-modality results in Table 4 reflect average performance over all possible cross-modality scenarios. The baseline and ConvLSTM performed poorly with F1-scores of 7.8% and 8.1% in PAMAP2, 9.3%, and 8.2% in DAS, and 16.2% and 12.8% in Smartsock. This demonstrates a highly diverse distribution of data across sensors of different modalities. The DirectMap approach achieved 40.4%, 44.8%, and 66.0% F1-score for PAMAP2, DAS, and Smartsock datasets, respectively. ActiLabel outperformed DirectMap by 19.3%, 21.4%, and 6.7% for PAMAP2, DAS, and Smartsock, respectively.

#### 6.3.2. Cross-Location Transfer

We examined transfer learning among chest, ankle, hand, arms, legs, and torso. The cross-location results in Table 4 represent the average values over all possible transfer scenarios. The baseline and ConvLSTM methods achieved F1-scores of 14.3% and 12.7% for the PAMPA2 dataset, respectively. Similarly, the baseline and ConvLSTM algorithms achieved 13.2% and 12.4% F1-scores, respectively, for DAS dataset. The relatively low F1-scores of the baseline and ConvLSTM algorithms can be explained by the high level of diversity between the source and target domains during cross-location. The DirectMap and ActiLabel both outperformed the baseline and ConvLSTM models, specifically, DirectMap and ActiLabel 63.4% and 70.8% F1-scores for PAMAP2, respectively, and 60.7% and 68.4% F1-scores for DAS.

#### 6.3.3. Cross-Subject Transfer

For this particular experiment, we included only four subjects from each dataset because there were only four subjects who performed all the activities in the protocol of the datasets. The baseline and ConvLSTM achieved 65.8% and 61.9% F1-score for PAMAP2, 67.1% and 56.8% F1-score for DAS, and 59.8% and 61.8% F1-score for Smartsock datasets. The baseline feature-based classifier achieved slightly higher performance than deep ConvLSTM. This can be explained by the fact that complex deep learning models may not be superior to feature-based algorithms when applied to data with low-dimensional feature space. Such deep learning models have been shown superiority to feature-based estimation models when adopted to datasets with high-dimensional channels (e.g., >100). However, the datasets used for our analysis had few channels of data from a few locations and sensors.

The DirectMap approach and ActiLabel obtained F1-scores of 85.4% and 82.7% in PAMAP2, 77.59% and 82.6% in DAS, and 82.6% and 77.5% in Smartsock, respectively. All the algorithms achieved higher F1-score values than the cross-location and cross-modality scenarios. This observation suggests that cross-subject transfer learning is an easier task to accomplish compared to cross-modality and cross-location because of the lower amount of variation in the distribution of the sensor data during cross-subject learning. These results suggest that data variations among different subjects can be normalized using techniques such as feature scaling, and feature selection before classification.

## 7. Discussions and Future Work

In this section, first, we discuss our work from several perspectives and discuss promising directions that will overcome some of the limitations of our work.

First, from the transfer learning perspective, the performance of different transfer learning algorithms depends on four factors. First, how well the target can distinguish between different physical activities when some correct labels are available. Second, how pure observations in target and source domains could be clustered into activity labels. Third, the accuracy of mapping between the source and target core clusters. Lastly, the capability of the source dataset in distinguishing between different activities when some labels are available. Table 4 shows that ActiLabel obtained an average F1-score of 59.3% in activity recognition of the PAMAP2 dataset, compared to 66.2% and 72.7% F1-scores for the DAS and Smartsock datasets, respectively. The collection of more diverse sensor modalities such as accelerometer, gyroscope, magnetometer, orientation, temperature, and heart rate, which are less representative of human physical activity events, affects every step in Actilabel, including core cluster identification, min-cost mapping, and activity recognition. As shown in Table 3, the strongest baseline classifier (e.g., 5-NN) achieved 78.9% average F1-score in detecting the activities from different sensor modalities from the PAMAP2 dataset; 5-NN reached a 94.5% activity recognition F1-score; and random forest obtained a 89.0% average F1-score for sensor modalities in DAS and Smartsock datasets, respectively.

Second, from the structural perspective, we note that the community detection-based algorithms outperform clustering algorithms in our setting. From Figure 8, we can observe that fast greedy, Lovain–Ward, and label propagation community detection algorithms obtained NMI of 0.16–0.51 and purity of 0.25–59 for PAMAP2, DAS, and Smartsock datasets, respectively, while the clustering methods, including affinity propagation, mean shift, and DBSCAN, achieved NMI of 0.42–0.70 and purity of 0.62–0.78 for these datasets, respectively. CCI, which is proposed as an extension to the community detection algorithms, achieved up to 20.4% higher NMI and 17.5% purity than these techniques. These results suggest that community detection algorithms are more reliable in the unsupervised clustering of datasets, in particular, human physical activity, when the models do not have prior knowledge on the number of the clusters. Although the clustering algorithms, such as affinity propagation and mean shift, eliminate the need to specify the number of clusters, they have other parameters, such as “preference” and “damping” for affinity propagation and “bandwidth for mean shift, that are challenging to optimize [43,44]. We note that tuning the structure of the input graph (e.g., modifying k for K-NN graphs) and merging strongly connected communities again, as proposed in CCI, improves the clustering quality comparing to the other community detection algorithms such as label propagation.

Finally, from the machine learning viewpoint of the activity recognition, we discuss the problem of poor performance of the baseline models (e.g., 31.6% F1-score, as shown in Table 4). Specifically, in the cross-modality scenario, the gap between the baseline and other transfer learning methods is the highest (e.g., gap of 32.6% to 59.9% in F1-score). One explanation is that the features adopted different distributions across different domains. We note that ConvLSTM did not meet the expectations in solving the problem of cross-domain transfer learning; the main reason that ConvLSTM could not improve the performance (e.g., 29.3% F1-score) of the baseline was an inadequate amount of data as the deep neural networks acquire a considerable amount to data to extract effective features through the deep convolution layers [45]. We believe that adding more data to the training dataset will improve the performance of the baseline method. Overall, assuming a lower F1-score for the baseline represents higher diversity between domains and, therefore, a more challenging transfer scenario, the cross-modality with 40.4–72.7% F1-score for DirectMap and ActiLabel is the most challenging transfer learning scenario. Overall, assuming a lower F1-score for the baseline represents higher diversity between domains and, therefore, a more challenging transfer scenario, the cross-modality with a 40.4–72.7% F1-score for DirectMap and ActiLabel is the most challenging transfer learning scenario.

There are few limitations to the evaluation process of the ActiLabel. First, we assume that the target activity labels are a subset of ones in the source domains. However, there are cases in real-word settings in which some of the activities in the target are not known to the source. The straightforward solution to this scenario is to add dummy nodes in the construction of bipartite graphs for the domain with fewer activities (e.g., source domain). However, such a solution is naive and results in mapping the dummy nodes from the source to the nodes associated with unknown activity labels from the target domain in the best case. To solve this issue, our ongoing work involves investigating practical approaches that allow for more complex mapping scenarios such as many-to-many mappings that capture all possible complex mapping situations that might occur in real-world and uncontrolled settings. Second, graph-based algorithms such as ActiLabel might encounter scalability challenges when deployed in large real-world datasets. We are planning to investigate the efficacy of replacing the k-NN graph with less computationally expensive graph structures such as kd-graphs and minimum spanning trees to enhance the scalability of the ActiLabel. Finally, the practical challenges of deploying our system in a real-world scenario will provide valuable information on the applicability of ActiLabel and help us improve our system. Therefore, one interesting future direction is the optimization of various computational components of ActiLabel for time, power, and memory efficiency given the dynamics of real-world scenarios.

Based on our analysis, Table 5 illustrates the merits and potential demerits of ActiLabels against analogous methods.

The aim of ActiLabel is to leverage the knowledge from a source domain where labeled data is abundant and use it to improve the performance of activity recognition task in a target domain where labeled data is limited. It is designed to handle transfer learning scenarios with different modalities, subjects, and sensor locations. The ActiLabel framework initiates community detection algorithms to identify core clusters of similar activities in the target domain and then maps them to corresponding activities in the source domain. By leveraging the relationships between activities and the knowledge from the source domain, ActiLabel aims to improve the activity recognition performance in the target domain. Additionally, ActiLabel’s performance is evaluated in three transfer learning setups: cross-modality transfer, cross-subject transfer, and cross-location transfer. These scenarios reflect the scope of application of ActiLabel in real-world situations where activity recognition needs to be performed across different sensor modalities, different individuals, and different sensor locations. While the focus of this study is activity recognition using wearable sensor data, the ActiLabel method’s underlying principles of transfer learning and community detection could potentially be applied to other domains and tasks where transfer learning deems fit. However, further research and experimentation would be needed to explore its effectiveness in those specific domains.

## 8. Conclusions

We introduced ActiLabel, a computational framework with combinatorial optimization methodologies for transferring physical activity knowledge across highly diverse domains. ActiLabel extracts high-level structures from sensor observations in the target and source domains and learns labels in the target domain by finding an optimal mapping between dependency graphs in the source and target domains. We showed that deep learning models and uninformed transfer learning techniques do not generalize well when transferring across different locations and sensor modalities, although their performance is acceptable in cross-subject learning. ActiLabel, however, provides consistently high accuracy for cross-domain knowledge transfer in various learning scenarios. Our extensive experimental results showed that ActiLabel achieves average F1-scores of 59.2%, 70.8%, and 82.7% for cross-modality, cross-location, and cross-subject activity recognition, respectively. These results suggest that ActiLabel outperforms the competing algorithms by 36.3%, 32.7%, and 9.1% in cross-modality, cross-location, and cross-subject learning, respectively.

## Figures and Tables

**Table 1:** Brief description on the datasets utilized for activity recognition.

Dataset	# Subject	# Activity	# Sample	# Feature	Sensors	Locations
PAMAP2	9	24	3,850,505	52	Accelerometer, Gyroscope, Heart rate monitor, Temperature, Orientation, Magnetometer	Chest, Hand, Ankle
DAS	8	19	1,140,000	45	Accelerometer, Gyroscope, Magnetometer	Left Arm, Right Arm, Left Leg, Right Leg, Torso
Smartsock	12	12	9888	30	Accelerometer, Stretch sensor	Chest

**Table 2:** Extracted time-domain features. E(.) represents the expected value of the input variable. Functions min(.),max(.),mean(.),median(.),tan(.), and size(.) compute the minimum, maximum, average, median, tangent, and size of an input vector, respectively.

Feature	Computation for Signal *S*
Peak amplitude of the signal	max(S)−mean(S)
Median of the signal	median(S)
Mean value of the signal	$\mu =\frac{\sum_{i=1}^{N}S_{i}}{N}$
Maximum value of the signal	max(S)
Minimum value of the signal	min(S)
Variance of the signal	$v=\frac{\sum_{i=1}^{N}{\left|s_{i}-\mu \right|}^{2}}{N-1}$
Standard deviation of the signal	$\sigma =\sqrt{\frac{\sum_{i=1}^{N}{\left|S_{i}-\mu \right|}^{2}}{N-1}}$
Root mean square of the signal	$\frac{\sum_{i=1}^{N}S_{i}^{2}}{N}$
Peak to peak difference	max(S)−min(S)
Zero crossing rate	$\frac{size(\left\{S_{i}|S_{i}==0,i=1,2,..,N\right\})}{N}$
Entropy of the signal	$-\sum_{i=1}^{N}S_{i}log\left(S_{i}\right)$
Skewness of the signal	$s=\frac{E{\left(S-\mu \right)}^{3}}{\sigma^{3}}$
Kurtosis of the signal	$k=\frac{E{\left(S-\mu \right)}^{4}}{\sigma^{4}}$
Mean magnitude of the signal	$M=\frac{\sum_{i=1}^{N}\sqrt{S_{i}x^{2}+S_{i}y^{2}+S_{i}z^{2}}}{N}$
Energy of the signal	$e=\sum_{i=1}^{N}S_{i}^{2}$
Range of the signal	r=max(S)−min(S)
Angle of the signal	$a=max(tan\left(\frac{S_{z}}{S_{x}^{2}+S_{y}^{2}}\right))$
Mean absolute deviation of the signal	$m=\frac{\sum_{i=1}^{N}\left|s_{i}-\mu \right|}{N}$

**Table 3:** Average F1-score(%) for activity classification over different sensor modalities and locations for PAMAP2, DAS, and Smartsock datasets.

Dataset	Type	k-NN	SVM	LR	RF	MLP	NB	QDA
PAMAP2	Modalities	78.9	65.6	65.5	**81.6**	73.6	55.0	65.7
	Locations	**93.8**	57.0	87.5	73.6	93.3	73.2	90.6
DAS	Modalities	**94.5**	75.7	86.4	93.1	87.7	69.5	88.9
	Locations	**97.1**	85.9	87.1	95.2	94.1	69.1	90
Smartsock	Modalities	83.7	74.6	65.3	**89.0**	71.8	59.5	62.8

**Table 4:** Activity recognition performance (F1-score).

Scenario	Dataset	Baseline	ConvLSTM	DirectMap	ActiLabel	Upper-Bound
Cross-modality	PAMAP2	7.8	8.1	40.4	**59.3**	80.8
	DAS	9.3	8.2	44.8	**66.2**	86.1
	Smartsock	16.2	12.8	66.0	**72.7**	84.2
Cross-location	PAMAP2	14.3	12.7	63.4	**70.8**	93.2
	DAS	13.2	12.4	60.7	**68.4**	89.8
Cross-subject	PAMAP2	65.8	61.9	**85.4**	82.7	98.1
	DAS	67.1	56.8	79.0	**80.3**	92.5
	Smartsock	59.8	61.8	**82.6**	80.0	95.2
**Average**		31.6	29.3	63.4	**71.9**	89.9

**Table 5:** Comparison of different transfer learning techniques.

Method	Advantages	Disadvantages
ActiLabel	- Leverages community detection algorithms - Graph-based modeling captures relationships between activities - Performs well in scenarios with similar sensor modalities and diverse source datasets	- Depends on availability of diverse sensor modalities - Scalability challenges with large datasets - Assumes target labels are subset of source domain labels
Deep Learning Models	- Can learn complex representations from raw sensor data - Strong performance with large labeled datasets - Can handle different modalities and adapt to domain shifts	- Requires large labeled datasets for training - Computationally expensive - May suffer from overfitting if training dataset is not representative
Uninformed Transfer Learning Techniques	- Simple and straightforward implementation - Applicable in scenarios with scarce labeled data - Provide a starting point for activity recognition - May not effectively leverage source domain knowledge - Do not adapt to domain shifts - Limited performance and applicability in diverse scenarios	

## Data Availability

Not applicable.

## References

[1] Piwek L., Ellis D.A., Andrews S., Joinson A. (2016). The rise of consumer health wearables: Promises and barriers. PLoS Med..

[2] Pantelopoulos A., Bourbakis N.G. (2010). A survey on wearable sensor-based systems for health monitoring and prognosis. IEEE Trans. Syst. Man Cybern. Part C.

[3] Esna Ashari Z., Ghasemzadeh H. Mindful Active Learning. Proceedings of the 2019 IJCAI conference (International Joint Conference on Artificial Intelligence).

[4] Esna Ashari Z., Chaytor N., Cook D.J., Ghasemzadeh H. (2020). Memory-Aware Active Learning in Mobile Sensing Systems. IEEE Trans. Mob. Comput..

[5] Ma Y., Esna Ashari Z., Pedram M., Amini N., Tarquinio D., Nouri-Mahdavi K., Pourhomayoun M., Catena R.D., Ghasemzadeh H. (2019). CyclePro: A Robust Framework for Domain-Agnostic Gait Cycle Detection. IEEE Sens. J..

[6] Hezarjaribi N., Esna Ashari Z., Frenzel J., Ghasemzadeh H., Hemati S. (2020). Personality Assessment from Text for Machine Commonsense Reasoning. arXiv.

[7] Pedram M., Rofouei M., Francesco F., Esna Ashari Z., Ghasemzadeh H. Resource-Efficient Computing in Wearable Systems. Proceedings of the 2019 IEEE International Conference on Smart Computing (SmartComp).

[8] Feichtenhofer C., Pinz A., Zisserman A. Convolutional two-stream network fusion for video action recognition. Proceedings of the IEEE Conference on Computer Vision and Pattern Recognition.

[9] Wang J., Zheng V.W., Chen Y., Huang M. (2018). Deep Transfer Learning for Cross-domain Activity Recognition. Proceedings of the 3rd International Conference on Crowd Science and Engineering.

[10] Cook D., Feuz K.D., Krishnan N.C. (2013). Transfer learning for activity recognition: A survey.

[11] Weiss K., Khoshgoftaar T.M., Wang D. (2016). A Survey of Transfer Learning.

[12] Pan S.J., Yang Q. (2009). A survey on transfer learning. IEEE Trans. Knowl. Data Eng..

[13] Fallahzadeh R., Alinia P., Ghasemzadeh H. Learn-on-the-go: Autonomous cross-subject context learning for internet-of-things applications. Proceedings of the 2017 IEEE/ACM International Conference on Computer-Aided Design (ICCAD).

[14] Wang J., Chen Y., Hu L., Peng X., Yu P.S. Stratified Transfer Learning for Cross-domain Activity Recognition. Proceedings of the 2018 IEEE International Conference on Pervasive Computing and Communications (PerCom).

[15] Fallahzadeh R., Esna Ashari Z., Alinia P., Ghasemzadeh H. (2021). Personalized Activity Recognition using Partially Available Target Data. IEEE Trans. Mob. Comput..

[16] Ordóñez F., Roggen D. (2016). Deep convolutional and lstm recurrent neural networks for multimodal wearable activity recognition. Sensors.

[17] Zhu G., Zhang L., Shen P., Song J. (2017). Multimodal gesture recognition using 3-D convolution and convolutional LSTM. IEEE Access.

[18] Yang Q. (2017). When Deep Learning Meets Transfer Learning. Proceedings of the 2017 ACM on Conference on Information and Knowledge Management.

[19] Chen J., Fang H.R., Saad Y. (2009). Fast approximate kNN graph construction for high dimensional data via recursive Lanczos bisection. J. Mach. Learn. Res..

[20] Maier M., Luxburg U.V., Hein M. Influence of graph construction on graph-based clustering measures. Proceedings of the Advances in Neural Information Processing Systems.

[21] Ferreira L.N., Zhao L. (2016). Time series clustering via community detection in networks. Inf. Sci..

[22] Puxeddu M., Petti M., Pichiorri F., Cincotti F., Mattia D., Astolfi L. Community detection: Comparison among clustering algorithms and application to EEG-based brain networks. Proceedings of the 2017 39th Annual International Conference of the IEEE Engineering in Medicine and Biology Society (EMBC).

[23] Blondel V.D., Guillaume J.L., Lambiotte R., Lefebvre E. (2008). Fast unfolding of communities in large networks. J. Stat. Mech. Theory Exp..

[24] Barton T., Bruna T., Kordik P. (2019). Chameleon 2: An Improved Graph-Based Clustering Algorithm. Acm Trans. Knowl. Discov. Data.

[25] Yan J., Yin X.C., Lin W., Deng C., Zha H., Yang X. A short survey of recent advances in graph matching. Proceedings of the 2016 ACM on International Conference on Multimedia Retrieval.

[26] Kuhn H.W. (1955). The Hungarian method for the assignment problem. Nav. Res. Logist. Q..

[27] Fisher M.L., Jaikumar R., Van Wassenhove L.N. (1986). A multiplier adjustment method for the generalized assignment problem. Manag. Sci..

[28] Clauset A., Newman M.E., Moore C. (2004). Finding community structure in very large networks. Phys. Rev. E.

[29] Reiss A., Stricker D. Introducing a new benchmarked dataset for activity monitoring. Proceedings of the 2012 16th International Symposium on Wearable Computers.

[30] Barshan B., Yüksek M.C. (2014). Recognizing daily and sports activities in two open source machine learning environments using body-worn sensor units. Comput. J..

[31] Fallahzadeh R., Pedram M., Ghasemzadeh H. Smartsock: A wearable platform for context-aware assessment of ankle edema. Proceedings of the 2016 38th Annual International Conference of the IEEE Engineering in Medicine and Biology Society (EMBC).

[32] Mannini A., Sabatini A.M. (2010). Machine learning methods for classifying human physical activity from on-body accelerometers. Sensors.

[33] Saeedi R., Schimert B., Ghasemzadeh H. (2014). Cost-sensitive feature selection for on-body sensor localization. Proceedings of the 2014 ACM International Joint Conference on Pervasive and Ubiquitous Computing: Adjunct Publication.

[34] McInnes L., Healy J., Melville J. (2018). Umap: Uniform manifold approximation and projection for dimension reduction. arXiv.

[35] Rendón E., Abundez I.M., Gutierrez C., Zagal S.D., Arizmendi A., Quiroz E.M., Arzate H.E. A comparison of internal and external cluster validation indexes. Proceedings of the 5th WSEAS International Conference on Computer Engineering and Applications.

[36] Powers D.M. (2020). Evaluation: From precision, recall and F-measure to ROC, informedness, markedness and correlation. arXiv.

[37] Brodersen K.H., Ong C.S., Stephan K.E., Buhmann J.M. The balanced accuracy and its posterior distribution. Proceedings of the 2010 20th International Conference on Pattern Recognition.

[38] Dueck D. (2009). Affinity Propagation: Clustering Data by Passing Messages.

[39] Comaniciu D., Meer P. (2002). Mean shift: A robust approach toward feature space analysis. IEEE Trans. Pattern Anal. Mach. Intell..

[40] Schubert E., Sander J., Ester M., Kriegel H.P., Xu X. (2017). DBSCAN revisited, revisited: Why and how you should (still) use DBSCAN. Acm Trans. Database Syst..

[41] De Meo P., Ferrara E., Fiumara G., Provetti A. Generalized louvain method for community detection in large networks. Proceedings of the 2011 11th International Conference on Intelligent Systems Design and Applications.

[42] Cordasco G., Gargano L. Community detection via semi-synchronous label propagation algorithms. Proceedings of the 2010 IEEE International Workshop on: Business Applications of Social Network Analysis (BASNA).

[43] Derpanis K.G. (2005). Mean shift clustering. Lect. Notes.

[44] Wang K., Zhang J., Li D., Zhang X., Guo T. (2008). Adaptive affinity propagation clustering. arXiv.

[45] Goodfellow S.D., Goodwin A., Greer R., Laussen P.C., Mazwi M., Eytan D. (2018). Atrial fibrillation classification using step-by-step machine learning. Biomed. Phys. Eng. Express.

